# Factors predicting medication adherence among Omani patients with chronic diseases through a multicenter cross-sectional study

**DOI:** 10.1038/s41598-023-34393-4

**Published:** 2023-05-01

**Authors:** Huda Al-Noumani, Maryam Alharrasi, Eilean Rathinasamy Lazarus, Sathiya M. Panchatcharam

**Affiliations:** 1 Adult Health and Critical Care Department, College of Nursing, Sultan Qaboos University, Muscat, Oman; 2 Research Section, Oman Medical Specialty Board, Muscat, Oman

**Keywords:** Health care, Medical research

## Abstract

Management of chronic diseases is complex and requires a long-term commitment to therapeutic medications. However, medication adherence is suboptimal. There is limited understanding of factors predicting medication adherence in chronic diseases in Oman. This study aimed to examine predictors of medication adherence (i.e. patient clinical and demographic data, patient-physician relationship, health literacy, social support) among Omani patients with chronic diseases. This study used a cross-sectional correlation design. Data were collected from 800 participants using convenience sampling between December 2019 and April 2020. Arabic versions of the Brief Health Literacy Screening tool, Multidimensional Scale of Perceived Social Support, Patient-Doctor Relationship Questionnaire, and Adherence in Chronic Disease Scale were used to measure study variables. Descriptive statistics, independent *t* tests, one-way ANOVA, Pearson correlations, and multivariate linear regression were used for analysis. The study found that factors such as the patient-physician relationship, social support, disease duration, employment status, and medication frequency significantly predicted medication adherence. Medication adherence was higher among those who were unemployed, had a better patient-physician relationship, and greater social support. However, medication adherence was lower with longer disease duration and higher daily medication frequency. Additionally, medication adherence was positively associated with perceived social support and the patient-physician relationship, but not with health literacy. In conclusion, the study reveals that patient characteristics, social support, and patient-physician relationships are key factors in predicting medication adherence in patients with chronic diseases in the Middle East. It emphasizes the importance of improving these aspects, considering factors like employment status, disease duration, and medication frequency, and enhancing healthcare provider-patient relationships and social support systems to boost adherence.

## Introduction

Non-communicable diseases (NCDs) are responsible for 71% of all global deaths. Cardiovascular (CVD), cancer, diabetes, and chronic respiratory diseases account for 31%, 16%, 7%, and 3%, respectively^1^. In Oman, the mortality rate due to NCDs is 70%, and the risk for premature death among people aged 30–70 years is 18%^2^. Of all deaths, cardiovascular diseases account for 36%, cancer for 11%, diabetes for 8%, and chronic respiratory diseases for 2%, comparable to those reported globally^1^. Chronic diseases are best controlled with the proper use of medication and adherence to the therapeutic regimen. Therefore, medication adherence among patients with chronic disorders is crucial to managing illness and reducing associated costs^3^. Adherence helps manage chronic disease and control healthcare expenditures through a reduction in inpatient hospital stays, mortality, morbidity, and emergency visits^4^. Nevertheless, adherence to long-term medication in chronic diseases remains sub-optimal, especially in developing countries^5^. Poor adherence to medications may increase the prevalence and complications of chronic diseases and lead to poor health outcomes such as quality of life and increased healthcare costs^5–8^.

Multiple factors may influence patient adherence to medications. These factors could be related to the patient (e.g. beliefs, knowledge, health literacy, and social support), healthcare providers (e.g. provider-patient communication, inadequate explanation about medication benefits and harmful effects), or the health system (e.g. limited access to health by patients, and limited healthcare coordination)^5–7^. Among all factors affecting chronic disease adherence, health literacy, social support, and the patient-physician relationship are among the key determinants^8,9^. For example, poor health literacy has been associated with poor health outcomes, such as increased hospitalization and mortality rates^5^. On the other hand, people with better health literacy are well-informed about their health and management and are actively involved in health decisions^5^. Therefore, they are more likely to adhere to medications. Additionally, interventions used to improve health literacy have significantly improved medication adherence^10^. Similarly, social support can reduce stress, promote patients’ self-esteem, facilitate patients' coping, and improve adherence to therapeutic plans^11^. A good patient-physician relationship may contribute to better health outcomes and patient satisfaction^9^.

In Oman, little is known about the factors that promote or hinder medication adherence in patients with chronic diseases. However, generally, low knowledge levels and health beliefs are associated with poor adherence to medications in chronic diseases in Oman^12^. For example, health beliefs may affect patient compliance with hypertension medications and ultimately affect hypertension control^13^. Therefore, there is a pressing need to investigate factors that can better manage medication adherence in these chronic diseases. In conclusion, the discussion of medication adherence and its related factors is applicable to the Oman setting as it highlights the significance of addressing NCDs and their management. By understanding and addressing the key determinants of medication adherence, Oman can develop targeted interventions to improve health outcomes and reduce healthcare costs associated with chronic diseases.

## Materials and methods

### Research design

This cross-sectional study used a correlational design that included patients with chronic diseases.

### Setting

Participants were recruited from outpatient specialty clinics across ten governorates in Oman.

### Population

The study aimed to investigate whether health literacy, social support, the patient-physician relationship, and other demographic and clinical factors can predict medication adherence among patients with chronic diseases (i.e. hypertension (HTN), ischemic heart disease (IHD), heart failure (HF), asthma, chronic obstructive pulmonary disease (COPD), and diabetes (DM)).

### Inclusion and exclusion criteria

Subjects were eligible for participation if they were: (1) Omani and primarily diagnosed with at least one of the following chronic diseases for a minimum of 1 year (i.e. DM, HTN, IHD, HF, asthma, and COPD), (2) aged 18 years and above, (3) prescribed at least one medication for the management of these diseases for a minimum of 1 year, and (4) able to speak and understand Arabic or English. Participants were excluded if they (1) had any cognitive impairment, (2) presented to the facility with an acute episode of diseases that might hinder their participation, or (3) refused to participate.

### Sample size and technique

The sample size was calculated using GPower 3.1. Based on a small expected effect size of 0.02 using a multiple linear regression model with an alpha error of 5% and a power (1-beta) of 80% with the predictors, a sample size of 759 patients was required for this study. We recruited 800 participants using convenience sampling to account for the probability of missing data.

### Data collection procedure

In this study, research assistants (RAs) who were nurses were recruited for data collection and entry. A protocol explaining the procedure of screening, recruitment, consenting, and data collection and entry was developed to train the RAs. A convenience sampling method was used to recruit participants. Before or during the specialty clinic day, RAs used electronic medical records (EMRs) to screen participants with chronic diseases who came for their follow-up appointments. Then, staff nurses sought their initial agreement to participate and approached those who met the inclusion criteria. Upon agreement, RAs met with them and explained the study details. Written consent was obtained from participants who agreed to participate. Participants were asked to self-administer the questionnaires, which took 15 to 30 min to complete. RAs ensured participant privacy, confidentiality, voluntary participation, and their right to withdraw from the study at any data collection point without any harm.

### Study variables and measuring instruments

#### Demographic and clinical variables

Age, gender, marital status, education, monthly income, and occupation were collected. Clinical variables included the number and duration of chronic diseases, number and frequency of medications per day, number of hospitalizations in the past year due to one or more chronic diseases, and the number of emergency visits.

#### Health literacy

The Brief Health Literacy Screening Tool (BRIEF) was used to measure health literacy. BRIEF is a self-report that consists of four items. Items of BRIEF are rated on a five-point Likert scale; responses for items 1, 2, and 4 are rated from 1 = always to 5 = never, while responses to item 3 are rated from 1 = not at all to 5 = extremely. The BRIEF score is based on the sum of the four non-weighted items and can range from 4 to 20. A higher score indicates higher health literacy. The convergent validity of BRIEF has been established, and the BRIEF sensitivity was 79%^14^. The Cronbach’s alpha of BRIEF in this study was 0.58.

#### Social support

The Multidimensional Scale of Perceived Social Support (MSPSS), Arabic version, was used to measure social support. This scale was designed to assess the perception of social support from three primary sources (i.e. family, friends, and significant others). MSPSS consists of 12 items rated on a seven-point Likert scale (1 = very strongly disagree to 7 = very strongly agree). The total score of the MSPSS ranges from 7 to 84, with higher scores indicating higher perceived social support. The reported Cronbach’s alpha coefficients for the MSPSS subscales of significant others, family, and friends were 0.91, 0.87, and 0.85, respectively. MSPSS’s validity was established by the original developer^15^. This scale has also been validated in an Arabic-speaking population^16^. In this study, the Cronbach’s alpha of MSPSS was 0.87. The reliabilities of the sub-scales were 0.79 for significant others, 0.75 for family support, and 0.89 for friend support.

#### Patient-physician relationship

A patient-doctor relationship questionnaire (PDRQ-9), Arabic version, was used to measure the patient-physician relationship^17^. This questionnaire was developed for use in primary care settings. The scale consists of nine items scored on a five-point Likert scale (1 = not at all appropriate to 5 = totally appropriate). The total score can range from 9 to 45, with a higher score indicating a more favorable relationship. The validity of PDRQ-9 has been established, and the scale’s reliability is excellent (Cronbach’s alpha of 0.94)^17^. In this study, the Cronbach’s alpha of the PDRQ-9 was 0.93.

#### Adherence to medications

“The Adherence in Chronic Disease Scale (ACDS), Arabic version, was used to measure medication adherence among patients with chronic disease. Initially, the scale was developed by Kubica and consisted of seven questions. Questions 1–5 assess patient behavior toward medication, while questions 6–7 assess factors indirectly affecting medication adherence. Answers to the seven questions are rated on a 0-4 point scale. Total scores vary from 0 to 28, with a higher score indicating better adherence. The scale was validated among patients with coronary artery disease (Aldona et al.^18^; Kosobucka et al.^19^), and the reported Cronbach’s alpha was 0.75 (Buszko et al.^20^). In this study, the ACDS Cronbach’s alpha was 0.93. The BRIEF, PDRQ-9, and ACDS were translated into Arabic, and permission to use and translate them was obtained from the primary developer. The translation was done following the WHO processes of translation and adaptation of instruments (World Health Organization^21^)”.

#### Data analysis

Collected data were analyzed using IBM SPSS Statistics version 25.0 (IBM Corp. Released 2017. IBM SPSS Statistics for Windows, Version 25.0. Armonk, NY: IBM Corp.). Continuous outcome variables were compared with categorical variables using independent t-tests or one-way ANOVA tests. Correlations were tested using the Pearson correlation. Statistically significant factors were taken into the multivariate linear regression model to adjust for the confounding factors. The homoscedasticity of the data was tested using normal probability plots, and collinearity statistics were tested using the variation inflation factor (VIF). A p-value of < 0.05 was considered statistically significant.

### Ethical approval and consent to participate

Ethical approval was obtained from the Research and Ethical Review and Approval Committee in the Ministry of Health, Oman (RC/RGCON/AHCC/18/01). The study conforms to the recognized standards of the declaration of Helsinki.

## Results

The study included 800 patients, with 74% of them aged 46 years and above and 51.9% female. Nearly two-thirds of them had a low education level (65.1%), and more than three-fourths were married (76.5%). Around half of them (52.1%) had a low monthly income (< 300), and 58.4% were unemployed. In addition, the majority had HTN and DM (43.8% and 37.4%, respectively), 58.1% had a single chronic disease, 30.8% took two medications or fewer in total, and only one-fourth (25.4%) took their pills once a day. The mean total score of health literacy was 11.89 (SD = 4.24), social support was 32.58 (SD = 3.84), the patient-physician relationship was 33.14 (SD = 7.55), and adherence was 23.83 (SD = 4.26) (Table 1).

**Table 1:** Distribution of demographic and clinical information (n = 800).

Variable	N (%)	Mean (SD^†^)	Min–Max
Age (years)			
≤ 45	203 (25.4)		
46–65	355 (44.4)		
> 65	242 (30.3)		
Gender			
Male	385 (48.1)		
Female	415 (51.9)		
Education status			
No/low education	521 (65.1)		
High school & above	279 (34.9)		
Marital status			
Single	71 (8.9)		
Married	612 (76.5)		
Divorced/widowed	117 (14.6)		
Monthly individual income (OMR^‡^)			
< 300	326 (52.1)		
301–1000	251 (40.1)		
> 1000	49 (7.8)		
Occupation			
Employed	155 (19.4)		
Retired	178 (22.3)		
Unemployed	467 (58.4)		
Chronic diseases			
Asthma	151 (18.9)		
COPD	121 (15.1)		
Hypertension	350 (43.8)		
Diabetes	299 (37.4)		
Ischemic heart disease	156 (19.5)		
Heart failure	157 (19.6)		
Number of diseases			
Single disease	465 (58.1)		
2 diseases or more	335 (41.9)		
Duration of diseases			
1–5 years	258 (32.4)		
6–10 years	234 (29.4)		
> 10 years	305 (38.3)		
Total number of medications			
Medication	246 (30.8)		
3–4 Medications	294 (36.8)		
> 4 medications	260 (32.5)		
Frequency of medications/day			
1 time	203 (25.4)		
2 times	407 (50.9)		
≥ 3 times	189 (23.7)		
Hospital admission in the past 1 year			
Yes	284 (35.5)		
No	516 (64.5)		
Emergency visits in the past 1 year			
Yes	361 (45.2)		
No	438 (54.8)		
Health literacy (BRIEF)		11.89 (4.24)	4–20
Patient-physician relationship (PDRQ-9)		33.14 (7.55)	9–45
Social support (MSPSS)		32.58 (3.84)	12–36
Medication adherence (ACDS)		23.83 (4.26)	5–28

^†^*SD* standard deviation, *OMR* omani rial, *Min–Max* minimum–maximum.

^‡^1 OMR = 2.60 US dollar.

In the bivariate analyses, statistically significant associations were observed between medication adherence and occupation (p = 0.018), the number of diseases (p = 0.004), daily frequency of medications (p = 0.001), disease duration (p = 0.05), and the number of hospitalizations (p = 0.04) and emergency visits (p = 0.004) in the past year (Table 2).

**Table 2:** Association between adherence and socio-demographic and clinical variables.

Variables	n (800)	Mean	SD^†^	p-value
Age (years)				
≤ 45	203 (25.4)	23.56	4.66	0.569
46–65	355 (44.4)	23.92	4.12	
> 65	242 (30.3)	23.93	4.12	
Gender				
Male	385 (48.1)	23.66	4.40	0.266
Female	415 (51.9)	23.99	4.13	
Education status				
No/low education	521 (65.1)	23.99	4.19	0.144
High school & above	279 (34.9)	23.53	4.38	
Marital status				
Single	71 (8.9)	22.87	5.16	0.138
Married	612 (76.5)	23.92	4.18	
Divorced/widowed	117 (14.6)	23.97	4.05	
Monthly income (OMR)				
< 300	326 (52.1)	23.77	4.37	0.482
301–1000	251 (40.1)	23.67	4.51	
> 1000	49 (7.8)	24.49	3.57	
Occupation				
Employed	155 (19.4)	23.73	4.14	0.018*
Retired	178 (22.3)	23.09	4.66	
Unemployed	467 (58.4)	24.15	4.11	
Number of diseases				
Single disease	498	24.18	4.19	0.004*
> 1 diseases	298	23.28	4.31	
Total medications				
1–2 medications	246	23.84	4.47	0.615
3–4 medications	294	24.00	4.10	
> 4 medications	260	23.64	4.25	
Frequency of medication/day				
1	203	24.64	3.39	0.001*
2	407	23.81	4.41	
≥ 3	189	23.00	4.64	
Maximum disease duration				
1–5 years	258	24.24	4.24	0.051*
6–10 years	234	23.98	4.15	
> 10 years	305	23.39	4.32	
Hospital admission in the past 1 year				
Yes	284	23.42	4.32	0.041*
No	516	24.06	4.22	
Emergency visits in the past 1 year				
Yes	361	23.49	4.22	0.042*
No	438	24.11	4.29	

^†^*SD* standard deviation, *OMR* omani rial.

*Significant at 0.05.

Significant values are in bold.

In addition, Pearson correlations showed significant and positive associations between medication adherence and perceived social support (r = 0.205; p < 0.001) and the patient-physician relationship (r = 0.276; p < 0.001), while no significant association was found with health literacy (r = 0.044; p = 0.216) (Table 3).

**Table 3:** Correlation between BRIEF, MSPSS, PDRQ, and ACDS.

Variable	n (800)	Correlation (r)	p-value
BRIEF	799	0.044	0.216
MSPSS	800	0.205	< 0.001*
PDRQ-9	800	0.276	< 0.001*

*BRIEF* brief health literacy screening tool, *MSPSS* multidimensional scale of perceived social support, *PDRQ*-*9* patient-doctor relationship questionnaire, *ACDS* adherence in chronic disease scale.

*Significant at 0.05.

Significant values are in bold.

All significant variables in the bivariate analyses were included in the regression model to examine the independent predictors of medication adherence. The multivariate linear regression model showed that the patient-physician relationship, social support, disease duration, occupation, and medication frequency significantly predicted medication adherence, and these factors explained about 14% of the variation in medication adherence (R2 = 0.14, F(6,793) = 21.67; p < 0.000). Medication adherence was significantly higher among unemployed participants (B = 0.76; p = 0.008), those with a higher patient-physician relationship (B = 0.15; p = 0.000), and better social support (B = 0.21; p = 0.000). In contrast, medication adherence was lower among participants who had a disease duration of more than 10 years (B = − 0.65; p = 0.027) and with a daily medication frequency of twice (B = − 0.86; p = 0.013) and three or more times (B = − 1.31; p = 0.002) (Table 4).

**Table 4:** Predictors of medication adherence using multiple linear regression^†^.

Variable	Unstandardized coefficients		Standardized coefficients	t	Sig^‡^	95.0% confidence interval
	B	SE	Beta			
Constant	12.64	1.37		9.25	0.000	9.96–15.32
PDRQ-9	0.15	0.02	0.27	8.16	0.000	0.12–0.19
MSPSS	0.21	0.04	0.19	5.57	0.000	0.13–0.28
Disease duration (> 10 years)	− 0.65	0.29	− 0.08	− 2.21	0.027	− 1.24– − 0.07
Occupation- unemployed	0.76	0.29	0.09	2.66	0.008	0.19–1.32
Medication frequency/day (≥ 3 times)	− 1.31	0.41	− 0.13	− 3.17	0.002	− 2.12– − 0.49
Medication frequency/day (2 times)	− 0.86	0.35	− 0.10	− 2.50	0.013	− 1.54– − 0.19

*MSPSS* multidimensional scale of perceived social support, *PDRQ*-9 patient-doctor relationship questionnaire.

^†^R2 = 0.141. This model used a stepwise elimination method. The model included variables: PRQ-9, MSPSS, occupation, disease duration, frequency of medications, hospitalization in the past 1 year, emergency visits in the past 1 year.

^‡^Table showed significant variables only.

## Discussion

This study investigated factors predicting medication adherence in common chronic diseases in Oman, namely, whether health literacy, social support, the patient-physician relationship, and other demographic and clinical characteristics can determine patient medication adherence. The key findings of this study were that unemployment, social support, and the patient-physician relationship were positive predictors of medication adherence among patients with chronic diseases in Oman. Additionally, the study revealed that taking medications more than once per day and a longer duration of the disease (> 10 years) predicted lower adherence among the study participants.

The current study showed that our participants demonstrated high social support; the mean total score was 32.58 out of a maximum score of 36, and about 76% were married. Higher social support is expected because most Omanis live in an extended family structure rather than nuclear families. This study revealed that participants with better social support demonstrated better medication adherence. This study is consistent with the literature on chronic diseases in different countries^22–25^. In Iran, a qualitative study among 34 patients with chronic conditions concluded that adherence to treatment regimens was higher among patients who received support from their spouses, family members, and friends^25^. Another survey among 2270 Chinese patients with chronic diseases reported that isolated and living alone people had low social support, resulting in low medication adherence; the same study showed that social support also mediates the relationship between adherence and loneliness^26^. Moreover, the specific type of social support provided has been linked to medication adherence. For instance, social support utilization, which is the extent to which the patient can utilize and receive the support, has been linked to better medication adherence among patients with diabetes^22^. Likewise, functional social support, which is the amount of emotional support, information, and encouragement a person gets from the surrounding social network, was linked to better medication adherence in hypertensive patients^24^. Patients with chronic diseases are at higher risk of developing depression and poor disease outcomes, and social support is linked to a reduction in depression and better health outcomes^27^. Therefore, strategies to improve social support should be implemented to reduce depression, increase medication adherence, and improve health outcomes for patients with chronic conditions. Healthcare providers, especially nurses, need to be aware that the quality of social support provided matters more than its quantity; hence, efforts to optimize social support quality should be accentuated in improving medication adherence.

The patient-physician relationship was one predictor of medication adherence in this study. We found that medication adherence was higher among participants with a stronger relationship with their physicians. This finding aligns with eastern, western, and Middle Eastern studies^9,28,29^. Consistent with our findings, a study reported that a high prevalence of non-adherence to medication was due to a lack of confidence in physicians and dissatisfaction with the relationship^29^. Another study reported that patients with a strong trust relationship with their providers showed better health-seeking behaviors and compliance with other therapeutic advice^30^.

Additionally, emphasizing the significance of medication adherence during patient-provider communication increases patients' positive experiences with medication and satisfaction^28^. The patient-provider relationship will be strengthened if healthcare providers are aware of a patient’s adherence level and barriers to adherence^31^. When healthcare providers seek information about patient adherence and related factors, they can design a personalized and culturally appropriate plan to improve medication adherence. Further, awareness of the adherence level will enhance trust in the relationship and encourage providers to provide education and involve the patient in decision-making related to their therapeutic regimen^31^. From the patient’s perspective, physicians’ knowledge of disease and medicine, listening skills, compassion, trustworthiness, shared decision-making, and time spent with the patients are critical factors leading to a successful patient-physician relationship^9^. Therefore, nurses should be aware of these factors while communicating with patients to empower medication adherence.

We found that medication adherence was significantly lower among patients who received medication more than once a day. Despite the inconsistency with some studies^32^, our finding is consistent with others^23,28^ and previous meta-analyses^33,34^. For example, one review revealed that patients with twice, three times, and four times daily dosing frequency reported 6% to 54% less adherence than those with a once-daily frequency^33,34^. Poor adherence with increased daily dosing frequency is expected because patients with chronic diseases have concurrent morbidities with multiple and complex long-term medications (polypharmacy). Accordingly, patients experience more side effects and stop taking medications within the first year^34,35^. In chronic diseases, polypharmacy remains a challenge to proper adherence, especially with increased age^34^. The prevalence of non-adherence in the elderly (> 65 years) with polypharmacy ranged between 6 and 55%^34^. Hence, researchers and providers should investigate the effects of polypharmacy on patient adherence and simplify medications in scheduling, dose fixing, and combination. Drug simplification and combinations are safe, efficacious, and result in a 26% or more reduction in non-adherence^3,34,35^.

The study found that patients with disease duration > 10 years had significantly lower adherence than those with a duration < 10 years. In contrast, some studies found no relationship^29^. A possible explanation for our finding is that prolonged disease duration lowers the adherence rate^33^. This could be related to long-term therapy, taking more medications with more frequency, which is consistent with our finding of the relationship between adherence and medication frequency. Another reason could be that patients with chronic diseases tend to develop depression, which lessens the adherence rate^36^. Nonadherence is two times higher in depressed patients than in non-depressed patients^36^. We also found that the unemployed had better adherence than employed and retired patients. The reason could be that employed participants have work commitments, making it challenging to deal with follow-up appointments compared to unemployed patients. Another possible basis for this finding is that 52% of participants were female, mostly housewives, and females are more involved in health-seeking behavior than men^37^. Our result is consistent with the findings of one review^38^ and inconsistent with another^39^, wherein other studies found no association^13,40^. Generally, the literature shows inconclusive findings in the link between disease duration, employment status, and medication adherence^32^. This variability can be attributed to heterogeneity in participants’ characteristics, belief systems, and disease perceptions across cultures and communities^32^, indicating that patients’ clinical and demographical characteristics could vary between different communities and should not be disregarded.

Health literacy is a factor receiving current attention for its impact on self-care management, knowledge about disease, health outcomes, self-efficacy, and medication adherence^41,42^. Unlike a study that reported a significant positive association between adequate health literacy and better medication adherence^10^, we found no significant association. However, our result is similar to another^42^. Our findings could be explained by variations in the tools used to measure health literacy and the diseases included, as we assessed patients with six types of chronic diseases. Another reason could be the decline in cognitive function, memory, and processing associated with aging and education^43^, as 65% of our participants had no or below high school education and 30% were aged above 65. However, Multiple studies have shown a significant association between low health literacy and poor medication adherence. Some of the main factors that contribute to this relationship include understanding prescription instructions, communication with healthcare providers, self-management skills, health beliefs and attitudes and socioeconomic factors. Improving health literacy can contribute to better medication adherence and, ultimately, improved health outcomes. Strategies to enhance health literacy include simplifying medication instructions, using plain language in patient education materials, enhancing communication between patients and healthcare providers, and implementing community-based interventions that support patients in managing their health^44–46^. Therefore, nurses, along with other clinicians, should focus on examining and planning effective educational, counseling, and behavioral strategies to improve health literacy among elderly and low-education patients.

Furthermore, the study’s findings on medication adherence among patients with chronic disorders and the factors influencing it have several implications for health policymakers in Oman like prioritize medication adherence, promote health literacy, strengthen social support systems, enhance patient-physician relationships, address healthcare system barriers, monitor and evaluate interventions and foster multi-sectoral collaboration. By considering these implications, health policymakers in Oman can develop targeted policies and interventions to improve medication adherence among patients with chronic diseases, ultimately leading to better health outcomes and reduced healthcare costs.

### Study limitations

This study has a few limitations; it was a cross-sectional and correlational study using a convenience sample that affects causal relationships and findings' generalizability. Future studies should be longitudinal and examine the effect of the study factors on adherence over time. Additionally, the findings of this study may only apply to outpatients. Hence, future studies might examine these factors in hospitalized patients. The study used a self-report measure of adherence that could introduce overestimation or recall bias. Furthermore, future studies might look at illness severity and depression concerning medication adherence in patients with chronic disease. Qualitative studies exploring social support preferences tailored to different cultures and societies are needed to improve medication adherence. The decision to focus on Omani patients in the study are several reasons, despite the diverse population in Oman. Some of these reasons include representativeness, cultural factors, social support systems and simplifying the study design. However, it is essential to note that focusing exclusively on Omani patients might limit the generalizability of the study's findings to other populations within Oman. Future research could consider including more diverse samples to better understand medication adherence and related factors among different cultural and ethnic groups in the country. This would help create a more comprehensive understanding of medication adherence and contribute to more inclusive healthcare policies and interventions.

## Conclusions

This study's strength is that it is the first study to examine patients' clinical and demographic variables, social support, the patient-physician relationship, and health literacy as factors predicting medication adherence across six types of chronic diseases. This study supports the importance of implementing educational courses for healthcare providers and students to emphasize methods to reinforce the significant role these factors play in improving medication adherence. Factors influencing medication adherence are multiple. Patient characteristics, social support, health literacy, and the patient-physician relationship are significant factors affecting medication adherence in patients with chronic disease. Importantly, due to the nature of chronic illnesses, the occurrence of comorbidities, and the complexity of their management, improving medication adherence should be the main aim of all healthcare providers, policymakers, patients, and families. Therefore, multidimensional strategies are required for effective improvement of medication adherence. Social support, health literacy, the patient-physician relationship, employment status, and the frequency of medications per day are all modifiable factors. They are essential to be studied and improved in patients with chronic diseases. Therefore, scientists, nurses, and other providers need to pay full attention to these factors and design appropriate strategies to help enhance medication adherence and overall health status.

## Data Availability

The dataset generated and/or analyzed during the current study are not publicly available due to participant privacy but are available from the corresponding author upon reasonable request.

## Abbreviations

NCDs: Non-communicable diseases
CVD: Cardiovascular
HTN: Hypertension
IHD: Ischemic heart disease
HF: Heart failure
COPD: Chronic obstructive pulmonary disease
DM: Diabetes
RAs: Research assistants
EMRs: Electronic medical records
BRIEF: The brief health literacy screening tool
MSPSS: The multidimensional scale of perceived social support
PDRQ: A patient-doctor relationship questionnaire
ACDS: The adherence in chronic disease scale
VIF: Variation inflation factor
